# The impact of health insurance on poverty among rural older adults: an evidence from nine counties of western China

**DOI:** 10.1186/s12939-021-01379-5

**Published:** 2021-01-25

**Authors:** Shaoguo Zhai, Shuiping Yuan, Quanfang Dong

**Affiliations:** 1 School of Public Administration, Northwest University, 1 Xuefu Road, Chang’ an District, Xi’ an, 710127 Shaanxi China; 2 Institute of Health Policy, Management and Evaluation, University of Toronto, 155 College Street, Toronto, ON M5T 3M6 Canada

## Abstract

**Background:**

Older adults are more prone to various diseases. Health insurance becomes effective mechanism to relieve financial burden when the insured is sick. In China, most older adults live in the countryside, and New Rural Cooperative Medical Scheme is a kind of health insurance system in rural areas. The relationship between New Rural Cooperative Medical Scheme and financial burden due to health expenditure of older adults in China was investigated. This paper aims at the impact of New Rural Cooperative Medical Scheme on the poverty among rural older adults.

**Methods:**

This study employs Probit model and Tobit model to assess the impact of New Rural Cooperative Medical Scheme on alleviating poverty among rural older adults based on a survey in nine representative counties in western China.

**Results:**

The findings show that diseases have significantly negative impact on rural elderly poverty. New Rural Cooperative Medical Scheme has impact on alleviating of the health-payment poverty due to catastrophic health expenditure, but the impact is limited. The impact of health insurance on poverty alleviation is greater for men, older adults aged between 60 to 69 and households in in economically poorer area than their counterparts.

**Conclusions:**

This study show the relationship between New Rural Cooperative Medical Scheme and catastrophic health expenditure of older adults in China. The results draw policy attention to introduce different reimbursement expense ratios for different groups to alleviate them from poverty based on more comprehensive insurance packages.

## Introduction

Poverty alleviation is a global issue because poverty is a constraint to the sustainable development of the economy and society. Poverty is categorized into two kinds: income-induced poverty which results from ability and resource constraints, and expense-induced poverty, which results from sudden events from external reasons such as diseases [1]. Chinese economic reform made huge contribution to alleviating income-poverty, but the expense-induced poverty needs to be focused. Disease risk is much of uncertainty, uncontrollability and irreversibility which reversely impede human capital. In this condition, people are less able to engage in economic activity so that they hardly escape poverty. Thus, expense-induced poverty is the biggest obstacle to poverty alleviation. According to The Chinese State Council Leading Group Office of Poverty Alleviation and Development, there were 18.47 million China rural adults who fell into poverty or were back to poverty again because of diseases in 2016, taking up 42.6% of rural poverty population. Globally, about 100 million patients fell into poverty because of high health expenditure to disease treatment and about 150 million people suffered catastrophic financial shock because of overspending health care [2]. Focusing on poverty is the key point to poverty alleviation for both China and other countries [3].

Unlike other groups, older adults are easily and badly affected by disease and become high-risk population of poverty due to their weak social roles and poor physical conditions [4]. More than 1 million older adults among 202 million older populations suffered at least one kind of chronic diseases in 2013 according to the China National Human Development Report issued in 2016. According to China Country Assessment Report on Aging and Health, compared to non-poor older adults, the poorer are more easily affected by poverty due to chronic diseases. In the condition of Chinese rural areas where family support and health care facilities are relatively lack, Chinese older adults living in rural areas are the targeted population most easily affected by poverty. Previous studies concluded that rural households having patients (aged over 60) with chronic diseases are three times as much likely to fall into disastrous poverty as other households due to high health expenses [5]. And about 31% of rural adults reported the difficulty to afford high health care fees [6].

One way to reimburse high expenses to health care is health insurance, which helps patients and families to overcome their financial burden [7]. Most of countries in the world have introduced health insurance in their healthcare system. China also established health insurance system in rural areas called New Rural Cooperative Medical Scheme (NRCMS). NRCMS is a “semi-mandatory” health insurance scheme that targets on population living in rural areas in China. It was established in 2003 and administered by the State Health and Family Planning Commission of China. This system intends to make healthcare more affordable for the rural poor. NRCMS is based on fee-for-service and cost-sharing, subsidized by both provincial and central governments. NRCMS covers expenses in all levels of public healthcare facilities, though the rate varies by region and by type of facilities. By 2008, more than 90% of the total population of China was enrolled [8].

Many studies focused on the effectiveness of NRCMS using different data source in China with inconsistent results. Whether the NRCMS helps to realize health equality or not and whether NRCMS has same impact on older adults who suffered disease as others remain uncertain. Jing S et al. [9] suggested that NRCMS reduce substantially financial burden for older adults with chronic diseases. Mateusz, Zhang and Chen [10] both found that low-income families benefit more from health insurance while Dai BJ [11] argued that the impact of health insurance is very limited for families suffered catastrophic expenditure due to chronic diseases. NRCMS as a compensation mechanism aiming at equality is even detrimental for low-income insured. Ma JD et al. [12] and Sagli G et al. [13] both thought that NRCMS could not cover most parts of health care expenditure for patients in rural areas. One study on NRCMS in 2018 indicated that the lower income and higher cost of health care co-incurred and together result to poverty and even liability in a long run [14], maintaining in a condition of “high demand, low utility, high burden, low income” [15]. Low-income families tend to give up necessary health treatments and this worsen their health condition, so they are easy to get in a vicious downward spiral between poverty and bad health [16, 17].

In the rapid aging society, we have to consider whether cost-sharing basis NRCMS is the effective way to reduce financial burden on health care. At present, the research on association of health expenditure with poverty among older adults in China is relatively lacking. Also, there is no study on the impact of NRCMS on poverty with updated database. Whether NRCMS plays important role in poverty reduction strategies needs to be explored based on updated data. This study is based on a new survey conducted in nine counties of western China in 2018 to assess the impact of NRCMS on poverty.

## Materials and methods

### Study settings

This study focuses on the impact of NRCMS on poverty alleviation. In particular, the problem of poverty caused by illness among older adults in western rural areas has a certain degree of representativeness and research significance. The overall development of rural areas in western my country is relatively backward, and the income level of residents is low. Also, the resources on the health service in the western rural areas are insufficient, and the supply and demand of medical and health resources are imbalanced compared to eastern China. Under the dual factors of economy and health, rural residents in the western region have a higher probability of suffering poverty. Our settings are the rural households in western counties.

### Recruitment and participants

Participants were recruited from July, 2018 to December, 2018, in nine representative counties within three western provinces in China.^1^ The rural population of 60 years old and above in 12 provinces in western was selected. We use stratified random sampling (two stages) methods. There were provinces selected at the first stage according to their average social and economic performance in recent 3 to 5 years (per capita GDP, Per Capita disposable income of residents). We selected Shaanxi, Guizhou and Gansu province. At the second stages, we selected counties according to their economic performance (per capita GDP and Per Capita disposable income of residents). The nine counties are: Langao county, Mei county and Shenmu county in Shaanxi Province; Huining county, Yuzhong county and Sunan county in Gansu Province; Yanhe county, Xishui county, Xiuwen county in Guizhou Province. We use the equation below to calculate the sample size which is 1200 with the consideration of design effect. We use the confidence level as 95% and the error tolerance as 0.4%. We sent out 1100 questionnaires and got back 1072 valid. The respondence rate is 97.4%.

1 $$ n=\frac{z^2\left[p\left(1-p\right)\right]}{e^2} $$

For each participant, a face-to-face interview was conducted by a trained investigator. This study was approved by the Ethics Committee of Northwest University, China. All participants provided written informed consent. We designed questionnaire to document 1072 participants’ family income, physical and mental conditions, current healthcare, healthcare expenditure, and also NRCMS reimbursement and its ratio.

### Reliability and validity

The reliability coefficient is 0.865, which suggests that reliability of this questionnaire is high. Content validity refers to the number of topics represented by the scale, and the correlation between each score and the total score can be used as an indicator of the validity of the questionnaire. The correlation coefficients for each category score and the total score were greater than 0.70 and were statistically significant, which means that the overall questionnaire content is valid.

### Variables definitions

Outcome variable: poverty or not, depth of poverty and intensity of poverty calculated by Foster-Greer-Thorbecke indices (FGT indices).

The Foster-Greer-Thorbecke indices aims to evaluate the degree of poverty by three dimensions: poverty or not, depth of poverty and intensity of poverty. As to the questions from reviewer, m is the poverty aversion coefficient which reflects FGT indices in different situations. When m is equal to 0, FGT is the incidence of poverty; when m is equal to 1, FGT is how far the household is away from poverty; when m is equal to 2, FGT is the intensity of poverty [18]. We employ FGT indices to measure identified poor population and their intensity of poverty and depth of poverty among rural older adults. The FGT equation as follows:

2 $$ {Poverty}_{mi}=\left\{\begin{array}{cc}{\left(\frac{z-{e}_i}{z}\right)}^m& {e}_i<z\\ {}0& {e}_i\ge z\end{array}\right. $$

In the equation, z represents poverty line (We chose average poverty line among nine counties which was 3328 yuan^2^), *e*_*i*_ represents household income per capita of older adult *i*. *e*_*i*_ ≥ *z* represents that *i* do not suffer poverty; *e*_*i*_ < *z* represents that *i* suffers poverty. When *m* = 0, *Poverty*_0*i*_ represents older adult *i* falling into poverty; When *m* = 1, *Poverty*_1*i*_ then represents the depth of poverty of older adult *i*; When *m* = 2, *Poverty*_2*i*_ represents the intensity of poverty of older adult *i*. This indicator is reflected in the questionnaire as: how much money did you get form NRCMS in the previous year? The reimbursement ratio is 39% on average. We use the question “how much money did you get from NRCMS in previous year?” to calculate the reimbursement ratio. Also, we set five ratio intervals (0% = 0; 0–20% = 1; 20–40% = 2; 40–60% = 3; 60–80% = 4; 80–100% = 5) for the convenience of data processing. We classified every respondent’s answer to the mentioned question into the intervals.

### Independent variable: catastrophic health expenditure and NRCMS

#### Catastrophic health expenditure

Measure of catastrophic health expenditure currently based on one of the following five ways: self-assessed health, the question that indicated if the participant is sick or is in hospital, whether the health payment of participant is over a fixed amount or a certain percentage of household income/expenditure. In fact, shock means that it has caused a certain impact, the self-assessed health clearly ignores this factor. And the participant in sick means he/she is at the risk of catastrophic health expenditure, which does not directly refer to catastrophic health expenditure. However, hospitalization itself is targeted at patients with severe disease. Therefore, once someone is in hospital, the family can be basically identified as suffering from catastrophic health expenditure. What’s more, hospitalization is only a kind of health service behavior, the essence behind it is the catastrophic impact of self-financing health expenses. The influence of the absolute amount of out-of-pocket expenditure varies from different income groups, the proportion of out-of-pocket expenditure can better reflect a family’s economic burden of disease relatively. Some studies on developing countries use health expenditures as 10% of consumer expenditures as the threshold for catastrophic health expenditures. Chinese scholars adopt this idea to use self-financing health expenses as a standard of 10% of total household income [19]. In view of the large number of missing values in the consumption data in this study, this paper measured the catastrophic health expenditure from the perspective of income. We also measured OP visits and hospitalization by self-reported questionnaire for supplement. The questions are: how many outpatient visits did you have in the previous year? How many times were you hospitalized in the previous year? Based on the above analysis, the catastrophic health expenditure is defined as the fact that older adults in the family were hospitalized or the health expenses for themselves reached 10% of the family annual income.

#### The NRCMS reimbursement

It is another independent variable which focuses on institutional policy. There are fewer residents who are not participating in health insurance, so the effect of poverty reduction simply by focusing on health insurance coverage is no longer applicable. Additionally, the welfare from health insurance is reflected by the reimbursement policy including reimbursement expense ratios, which are not decided by insured. Thus, it is not an endogenous variable [20]. We measure this variable by asking: How much is reimbursed by NRCMS (the proportion)? A higher level of health insurance coverage means a lower proportion of out-of-pocket health expenses for residents, and its anti-poverty effect is obvious in theories. On the contrary, the health care can release the demand for health services and even create the risk of overtreatment in reality [21]. In a nutshell, the impact of NRCMS reimbursement on poverty reduction is not clear yet.

### Control variables

We choose control variables from three dimensions: population structure, social resource, and family support. Population structure contains gender, age, education; social resource contains economic and social involvement indicating how many kinds of economic and social activities you participate; family support contains marital status, number of children, relationship with children, financial support from children, frequency of interaction with children by telephone, frequency of face-to-face interaction with children. Besides, the region are also defined as control variable in considering it’s difference in economic development and health conditions. All variables are shown in Table 1.

**Table 1:** Variables definitions

Variables	Definitions
Poverty	Fall in poverty = 1; Not fall in poverty = 0
Catastrophic health expenditure or not	The proportion of out-of-pocket expenses is over 10% of total household income per capita (Yes = 1, No = 0)
Reimbursement expense ratio	0% = 0; 0 ~ 20% = 1; 20 ~ 40% = 2; 40 ~ 60% = 3; 60 ~ 80% = 4; 80 ~ 100% = 5
Interaction term	Disease shock and reimbursement expense ratio
Martial status	With spouse =1; without spouse = 0
Number of children	Continuous variable
Relationship with children	Very not harmonious = 1; not harmonious = 2; average = 3; harmonious = 4; very harmonious = 5
Financial support from children	0 yuan = 0; 0 ~ 1500 yuan = 1; 1500 ~ 3000 yuan = 2; 3000 ~ 4500 yuan = 3; 4500 ~ 6000 yuan = 4; more than 6000 yuan = 5
Frequency of face-to-face interaction with children	At least once within 1 week = 1; at least once within 1 month = 2; at lease once within 3 months = 3; at least once within semi-year = 4; at lease once within 1 year = 5
Frequency of interaction by telephone with children	At least once within 1 week = 1; at least once within 1 month = 2; at lease one within 3 months = 3; at least once within semi-year = 4; at least once within 1 year = 5
Social involvement	Continuous variable indicating how many kinds of social activities you participate
Economic involvement	Continuous variable indicating how many kinds of economic activities you participate
Gender	Male = 1, female = 0
Age	Continuous variable
Years for education	Continuous variable

### Model setting

We used three indicators to measure poverty: poverty or not, depth of poverty and intensity of poverty. The depth of poverty reflects how far, on the average, the poor are from that poverty line. The intensity of poverty is to assess the extent to which the standard of living of the poor population is under the poverty line. These three measurements reflect poverty together which help us to further explain the impact of NRCMS on poverty. The results show the impact of NRCMS on depth of poverty and the impact of NRCMS on intensity of poverty. In this way, poverty or not is a binary variable, so we use Probit model to test its result. The latter two are continuous variables with values between 0 to1. When household income per capita is higher than poverty line mentioned above, the value be 0. The data is censored at left bound on the number line, so we use Tobit model to test their results. The model as follows:

3 $$ {Poverty}_{mi}={\alpha}_0+{\beta}_{1i}{Shock}_i+{\beta}_{2i}{Medi}_i+\gamma {x}_i+\varepsilon $$

4 $$ {Poverty}_{mi}={\alpha}_0+{\beta}_{1i}{Shock}_i+{\beta}_{2i}{Medi}_i+{\beta}_{3i}{Shock}_i\times {Medi}_i+\gamma {x}_i+\varepsilon $$

In these equation, *Poverty*_*mi*_ is outcome variable representing if participant falls into poverty or not, depth of poverty and intensity of poverty; *Shock*_*i*_ is the independent variable representing catastrophic health expenditure which mentioned above; *Medi*_*i*_ as another independent variable represents NRCMS reimbursement expense ratio; *x*_*i*_ represents control variables mentioned above; *ε* represents random disturbance term. Equation (3) is to test the hypothesis that if catastrophic health expenditure has impact on poverty. The eq. (4) is to test the hypothesis that MRCMS has impact on reducing negative effect of catastrophic health expenditure on poverty by introducing the interaction term between catastrophic health expenditure and reimbursement expense ratio.

## Results

### Descriptive statistical analysis

Among the whole samples (*N* = 1070), about 33% are identified as falling in poverty, about 32% are badly affected by disease, and 69% are reimbursed by NRCMS with 0.4 reimbursement ratio. It could be found that poor rural older adults are more easily and badly affected by disease compared with their non-poor counterparts and their reimbursement ratio also are higher than counterparts. From the family support dimension, poor rural older adults have more children than their non-poor counterparts, but the relationship with children, the frequency of face-to-face interactions, and the financial supports from children among non-poor participants are all better than their poor counterparts. From social resource dimension, the frequency of social and economic involvement for poor rural older adults is lower than that for their counterparts. From the population structure dimension, the data showed that compared with non-poor rural adults, poor rural adults are identified with more women, higher age, lower education, and smaller proportion of spouse. To some extent, this indicates the resources possessed by the poor elderly in rural areas are most inferior to those possessed by the non-poor elderly, which provides intuitive empirical evidence for the next regression analysis. Table 2 shows the descriptive statistical analysis.

**Table 2:** Characteristics by poor group vs. Non-poor group

Variables	Total		Non-poor group Not falling into poverty		Poorer group Falling into poverty		T value
	Mean	SD	Mean	SD	Mean	SD	
Poverty	0.33	0.47	–	–	–	–	–
Catastrophic health expenditure or not	0.32	0.46	0.29	0.45	0.36	0.48	−2.41^***^
Reimbursement ratio	2.37	1.82	2.32	1.83	2.5	1.79	−1.04
Martial status	0.72	0.45	0.72	0.44	0.71	0.45	0.33
Number of children	3.24	1.49	3.10	1.46	3.50	1.48	−4.21^***^
Relationship with children	4.03	0.84	4.11	0.81	3.87	0.87	4.38^***^
Financial support from children	1.62	1.64	1.78	1.76	1.32	1.30	4.70^***^
Frequency of face-to-face interaction with children	1.97	1.37	2.06	1.40	1.79	1.27	3.17^***^
Frequency of interaction by telephone with children	1.56	1.03	1.56	1.04	1.53	0.99	0.48
Social involvement	2.00	0.95	2.08	0.97	1.85	0.87	3.85^***^
Economic involvement	0.55	0.63	0.57	0.64	0.51	0.62	1.60
Gender	0.58	0.49	0.61	0.48	0.52	0.50	2.78^***^
Age	70.67	7.56	69.88	7.28	72.12	7.73	−4.64^***^
Years of education	3.41	3.57	3.92	3.72	2.42	3.02	6.55^***^

Note:①T value means Statistical significance test result of variable difference between poorer group and non-poor group; ②***, **, * respectively means the significance level of 0.01, 0.05 and 0.1

### Regression analysis

#### The impact of catastrophic health expenditure on poverty among rural older adults

Table 3 shows that the catastrophic health expenditure had significant positive effect on poverty, depth of poverty, and intensify of poverty (99% CI). This confirmed previous studies. Rural older adults with catastrophic health expenditure (their health payment took up more than 10% of total income or in hospital) were more likely to great degree of depth of poverty, which indicated that catastrophic health expenditure significantly increase degree of poverty including depth and intensity of poverty. NRCMS reimbursement expense ratio is not significantly associated with poverty among rural older adults.

**Table 3:** The impact of NRCMS reimbursement on poverty

	Poverty or not	Depth of poverty	Intensity of poverty
Catastrophic health expenditure or not	0.1734^***^ (0.1455)	0.2450^***^ (0.0644)	0.1743^***^ (0.0461)
Reimbursement expense ratio	−0.0115 (0.0370)	− 0.0161 (0.0171)	− 0.0129 (0.0122)
Martial status	0.0164 (0.1582)	0.0129 (0.0680)	0.0092 (0.0485)
Number of children	0.0286^*^ (0.0482)	0.0269 (0.0207)	0.0179 (0.0148)
Relationship with children	−0.0495^*^ (0.0811)	− 0.0513 (0.0364)	− 0.0307 (0.0260)
Financial support from children	−0.0606^***^ (0.0433)	− 0.0932^***^ (0.0212)	−0.0702^***^ (0.0153)
Frequency of face-to-face interaction with children	−0.0651^***^ (0.0674)	−0.0945^***^(0.0285)	− 0.0649^***^ (0.0203)
Frequency of interaction by telephone with children	0.0578^**^ (0.0831)	0.0841^***^ (0.0355)	0.0579^**^ (0.0254)
Social involvement	0.0183 (0.0702)	0.0279 (0.0348)	0.0224 (0.0249)
Economic involvement	0.0081 (0.1174)	0.0172 (0.0568)	0.0117 (0.0406)
Gender	−0.0317 (0.1370)	−0.0271 (0.0631)	− 0.0162 (0.0451)
Age	0.0021 (0.0112)	0.0049 (0.0048)	0.0039 (0.0034)
Years for education	−0.0038 (0.0202)	−0.0036 (0.0095)	− 0.0024 (0.0068)
Observations	520	520	520
Pseudo R^2^	0.2072	0.2402	0.3150

Note: coefficients show marginal effects based on mean; numbers in bracket are consistent standard errors. ***, **, * respectively means the significance level of 0.01, 0.05 and 0.1

### The impact of NRCMS on poverty

We introduced interaction term between catastrophic health expenditure and reimbursement expense ratio to assess the impact of NRCMS on poverty. The results indicated that the interaction term was negatively associated with poverty. Each 20% increase of reimbursement ratio reduced 6.51% for the probability of poverty. These findings suggested that NRCMS has positive effect on alleviating poverty among rural older adults. The column 3 and 4 in Table 4 showed that NRCMS reimbursement also had a significantly negative relationship with depth of poverty and intensity of poverty (90% CI). Every 20% increase of reimbursement ratio reduced depth of poverty by 8.94% and intensity of poverty by 6.38%. Besides, we also found that family support had great effect on alleviation of poverty. Non-rural older adults who got more family support such as more financial supports from children, more interaction with children, and harmony relationship with children had less degree of intensity and depth of poverty and lower probability of poverty.

**Table 4:** The impact of NRCMS reimbursement on health payment-induced poverty

	Poverty or not	Depth of poverty	Intensity of poverty
Catastrophic health expenditure or not	0.3145^***^ (0.2220)	0.4478^***^ (0.1016)	0.3192^***^ (0.0728)
Reimbursement expense ratio	0.0289 (0.0601)	0.0411 (0.0273)	0.0280 (0.0196)
Interaction term	−0.0651^***^ (0.0743)	− 0.0894^***^ (0.0335)	− 0.0638^***^ (0.0240)
Martial status	0.0262 (0.1596)	0.0243 (0.0678)	0.0175 (0.0484)
Number of children	0.0280^*^ (0.0477)	0.0256 (0.0206)	0.0170 (0.0147)
Relationship with children	−0.0472^*^ (0.0815)	−0.0466^***^ (0.0362)	− 0.0274 (0.0259)
Financial support from children	0.0602^***^ (0.0436)	−0.0919^***^ (0.0210)	−0.0693^***^ (0.0151)
Frequency of face-to-face interaction with children	−0.0710^***^ (0.0692)	−0.1016^***^ (0.0285)	− 0.0700^***^ (0.0204)
Frequency of interaction by telephone with children	−0.0627^**^ (0.0834)	0.0898 (0.0353)	0.0620^***^ (0.0253)
Social involvement	0.0148 (0.0709)	0.0228 (0.0345)	0.0188 (0.0247)
Economic involvement	0.0148 (0.0709)	0.0108 (0.0567)	0.0069 (0.0406)
Gender	−0.0488 (0.1373)	−0.0481 (0.0633)	− 0.0313 (0.0453)
Age	0.0022 (0.0112)	0.0051 (0.0048)	0.0040 (0.0034)
Years for education	−0.0023^***^ (0.0203)	−0.0014 (0.0095)	− 0.0008 (0.0068)
Observations	520	520	520
Pseudo R^2^	0.2170	0.2501	0.3266

Note: numbers in bracket are consistent standard errors. ***, **, * respectively means the significance level of 0.01, 0.05 and 0.1

### Robust test

We used 3380 yuan which was the average poverty line among nine counties to test hypotheses. For a more robust result, we also used 3300 yuan, which was the average poverty line across country in 2017 to test all hypotheses again and the results shown are consistent. This further indicates that NCMS can alleviate the negative effect of disease impact on rural poverty. Table 5 shows the results of robust test.

**Table 5:** Robust test

	Poverty or not	Depth of poverty	Intensity of poverty
Interaction term 2 (Catastrophic health expenditure or not and reimbursement ratio)	−0.0611^**^ (0.0741)	− 0.0861^***^ (0.0336)	− 0.0613^***^ (0.0240)
Observations	520	520	520

Note: numbers in bracket are consistent standard errors. ***, **, * respectively means the significance level of 0.01, 0.05 and 0.1

### Heterogeneity test

The marginal utility of health insurance varies by gender and age for different group population [22]. Besides, the health insurance policy, health resource supply and health services are diverse because of the difference of economic growth between areas. We further employ Probit and Tobit model to analyze how the impact of NRCMS on poverty differs across gender, age and economic development of areas, attempting to draw policy attention to different groups and areas.

#### The impact of NRCMS reimbursement expense ratio on poverty in different areas of economic development

Compared to rural older adults living in economically better area, rural older adults living in economically inferior and poor areas are more likely suffering poverty. Each 20% increase of reimbursement expense ratio reduced the negative effect of poverty by 43.24% for participants in economically inferior area and 31.36% participants in economically poor area, both higher than 5.44% for participants in economically better area.

The NRCMS had significantly positive effect on alleviating poverty as to the depth and intensity of poverty. A 20% increase of reimbursement expense ratio reduced the negative effect of catastrophic health expenditure on depth and intensity of poverty by 21.21% for participants living in economically inferior area and 14.98% for participants living in economically poor area, respectively compared with 2.06% for participants living in economically better area. Table 6 shows the results.

**Table 6:** The impact of NRCMS reimbursement on health payment-induced poverty in different areas of economic development

	Poverty or not			Depth of poverty			Intensity of poverty		
	economically better area	economically inferior area	economically poor area	economically better area	economically inferior area	economically poor area	economically better area	economically inferior area	economically poor area
Catastrophic health expenditure or not	−0.0193 (0.3098)	0.7997*** (0.2597)	0.4027** (0.2029)	0.0756 (0.1770)	0.4690*** (0.1322)	0.1239 (0.0918)	0.0688 (0.1057)	0.3409*** (0.0953)	0.0708 (0.0721)
Interaction term	−0.0544 (0.1560)	−0.4324*** (0.1434)	− 0.3136*** (0.1171)	−0.0206 (0.0921)	− 0.2121*** (0.0779)	−0.1498*** (0.0489)	− 0.0206 (0.0921)	−0.2121*** (0.0779)	− 0.1498*** (0.0489)

Note: numbers in bracket are consistent standard errors. ***, **, * respectively means the significance level of 0.01, 0.05 and 0.1

#### The impact of NRCMS reimbursement expense ratio on poverty based on gender

The impact of NRCMS on poverty differed by gender. The NRCMS had more positive effect on alleviating poverty among males than females. A 20% increase of NRCMS reimbursement expense ratio reduced the negative effect of catastrophic health expenditure on poverty is 46.22% for males and 8.34% for females. A 20% increase of NRCMS reimbursement expense ratio reduced depth and intensity of poverty by 24.04% for males and 6.36% for females. Table 7 shows the impact of NRCMS on poverty based on gender.

**Table 7:** The impact of NRCMS reimbursement on health payment-induced poverty Based on gender

	Poverty or not		Depth of poverty		Intensity of poverty	
	Male	Female	Male	Female	Male	Female
Catastrophic health expenditure or not	0.5009*** (0.1827)	0.3922** (0.1937)	0.2687*** (0.1044)	0.2219** (0.1054)	0.2687*** (0.1044)	0.2219** (0.1054)
Interaction term	−0.4622*** (0.1145)	− 0.0834 (0.0988)	−0.2404*** (0.0627)	− 0.0636 (0.0549)	−0.2404*** (0.0627)	− 0.0636 (0.0549)

Note: numbers in bracket are consistent standard errors. ***, **, * respectively means the significance level of 0.01, 0.05 and 0.1

#### The impact of NRCMS reimbursement expense ratio on poverty based on age groups

The impact of NRCMS on poverty showed differences in different age groups. This impact is much greater among young-old (60–69) age groups than other groups. A 20% increase of NRCMS reimbursement expense ratio reduced negative effect of catastrophic health expenditure on poverty by 31.95% for aged 60–69 group and by 9.74% for aged group over 79. A 20% increase of NRCMS reimbursement expense ratio reduced depth and intensity of poverty by 18.81% for aged 60–69 group and by 5.41% for aged group over 79. Table 8 shows the impact of NRCMS on poverty based on age groups.

**Table 8:** The impact of NRCMS reimbursement on health payment-induced poverty Based on age groups

	Poverty or not			Depth of poverty			Intensity of poverty		
	60–69	70–79	> 79	60–69	70–79	> 79	60–69	70–79	> 79
Catastrophic health expenditure or not	0.3975* (0.2119)	0.3383* (0.2037)	1.3204*** (0.4325)	0.2415** (0.1221)	0.1818* (0.1033)	0.5708*** (0.2262)	0.2415** (0.1221)	0.1818* (0.1033)	0.5708*** (0.2262)
Interaction term	−0.3195*** (0.1108)	−0.2492** (0.1121)	− 0.0974 (0.1901)	−0.1881*** (0.0635)	− 0.1287** (0.0567)	−0.0541 (0.1271)	− 0.1881*** (0.0635)	−0.1287** (0.0567)	− 0.0541 (0.1271)

Note: numbers in bracket are consistent standard errors. ***, **, * respectively means the significance level of 0.01, 0.05 and 0.1

## Discussion

This study aims at the health insurance in rural China, NRCMS, to analyze whether this scheme is an effective way to reduce poverty among rural older adults with disease. We discussed findings from four points as follows:

The catastrophic health expenditure brought about higher probability of poverty and degree of depth and intensity of poverty which confirms the previous research [23–25]. A number of previous studies have shown that, the catastrophic health expenditure not only resulted in the reduction of productivity and income [4, 26, 27], but also increased the health care expenditure. Limited by budget constraint, people tend to spend money on daily expenditure rather than ability investment, this dilemma was detrimental for families’ financial gains [27, 28]. Even though many rural families were willing to take loans to solve their current financial problems, this increases their long-term financial risk instead [29].

The NRCMS reduces the financial risk of poverty among rural older adults but the impact was limited, which could be explained in the following three points. First, the spread and development of NRCMS leads to inflation of health prices and thus decreases the impact of NRCMS on alleviating poverty [21]. Because of the demand elasticity for health care market, there exists an upward spiral between the price of health care and the insurance coverage leading to insurance market inflation. The insured react to the market to increase purchasing health services but not to reduce the health expenses [30]. This inflation stimulates welfare waste and moral hazard of health care suppliers and weakens the effectiveness of health care [31, 32]. The health care expenditure per capita in China rose up from 1314.26 yuan in 2009 to 3783.83 yuan in 2017 according to China Statistical Yearbook Database. And the proportion of health care expenditure per capita in consumption expenditure also rose from 31.09% in 2013 to 34.54% in 2017. Second, NRCMS focuses more on hospitalization than outpatient services for chronic diseases. The NRCMS contributes to financial protection for rural household against catastrophic diseases [8, 33], but research evidence implied that outpatient services and drugs were more important factors for poverty rather than hospitalization [34]. Patients with chronic diseases visited doctors frequently but their outpatient services fees and drugs were hardly reimbursed by NRCMS [8]. Yip and Hsiao [34] found that 11.6% of total rural families fell into poverty due to high outpatient services fees for chronic diseases. Under this circumstance, there are still 64.5% older adults concerning that they cannot afford health care fees [35]. Third, the NRCMS did not consider financial compensation for people who cannot earn livings due to their bad health conditions. They easily lose their ability to getting income after suffering diseases and falling into income-caused poverty again. In this case, they still cannot afford health care fees even if it is not much.

There exist differences of impact of NRCMS on poverty between different gender and age individuals. The impact of NRCMS on alleviating poverty is greater for males and young-old adults (age = 60–69) because they are more likely to go back labor market to earn their livings and more likely to struggled from poverty. Whether or not to seek health care usually involves a shared decision made by household members, in which the head of household plays a critical role. However, this finding is contrary to previous studies which have suggested that households headed by a female, an unemployed person or a person having little education were more likely to be beneficial from NRCMS [36, 37]. In a recent study, Ma [38] thought that the age of the head of household influenced the poverty level due to healthcare expenditure. As the age of the head of household increases, the risk of poverty increases by 1.83 percentage point. Thus, aging is one of the key factors impeding poverty alleviation. Some social support such as raising the government’s high co-payments and strengthening the policy inclination of older adults could be considered with China stepping into aging society. To achieve poverty alleviation, policy makers could accurately target the characteristics of the poor, capture the poverty-reducing characteristics at the individual, family, and institutional levels, prioritize vulnerability [39].

There also exists difference of impact of NRCMS on poverty between different economic development areas. The impact of NRCMS on alleviating poverty is greater for individuals living in economically poorer area than their counterparts. A study of catastrophic health expenditure in China [6] found that health care expenditure imposes a heavy financial burden on rural households. Guo [40] demonstrated significant regional differences in the rural poverty-stricken groups and gradually gather in the central and western regions over time in China between 1978 and 2014. The incidence of poverty due to high healthcare expenditure is significantly higher in the central and western regions than in the east, which is consistent with the distribution of poverty-stricken populations in China. For the area difference, Wang and Liu [41] also found that the impact of NRCMS on alleviating poverty is greater in central and western China where society and economy develop more slowly than eastern China. Ma [38] found that the economic development-focused approach to regional poverty alleviation has not reached the maximum point of convergence with China’s current demand for health. There are two factors that can explain: on the one hand, people living in eastern China focus more on healthcare so that they purchase more health goods and have more financial burden. The average number of health care visits per capita per year is 7.77 in eastern China, higher than 4.96 in western China, 4.81 in central China and 3.89 in Northeastern China. On the other hand, rural older adults in eastern China possess more types of assets so NRCMS works more slightly than other areas in China.^3^

This study has several limitations. The cross-sectional survey was conducted in a short term, so this only revealed the correlation between catastrophic health expenditure and depth of poverty and intensity of poverty. We are unable to predict the long-term impact of diseases on poverty. Extensive studies focusing on causal effect in a long term are warranted to assess the effectiveness of NRCMS in a more convincing way. This study only focused on poverty counties in western China. The results need to be generalized in other areas.

## Conclusions

Catastrophic health expenditure is a vital factor for falling into poverty among rural older adults. NRCMS has impact on alleviating of the health-payment poverty due to catastrophic health expenditure but the impact is limited. The impact of NRCMS on poverty alleviation is greater for men, young-old adults (age = 60 to 69) and households in in economically poorer region than their counterparts. The findings draw policy attention to disease prevention and health promotion. Older adults living in poor rural areas are needed to be focused to promote equality, accessibility, and equity of healthcare delivery.

## Data Availability

Please contact author for data requests.

## Footnotes

1 Samples from Langao county, Mei county and Shenmu county in Shaanxi Province; Huining county, Yuzhong county and Sunan county in Gansu Province; Yanhe county, Xishui county, Xiuwen county in Guizhou Province.

2 It is noted that this survey was based on household and we defined participant’s income as his/her household’s income. The poverty lines come from the average level of nine counties.

3 According to the results, in the economically better area, the negative impact of catastrophic health expenditure on rural elderly poverty is significantly less than the economically poorer area.Thus, we can guess that,compared with the economically poorer area,in the economically better area, other forms of capital, other than health insurance, have played a major role in poverty alleviation in eastern China.
